# Multi-omics technologies: Novel tools and methods for assessing nerve injury and regeneration

**DOI:** 10.4103/NRR.NRR-D-25-00610

**Published:** 2025-10-30

**Authors:** Qiang Zhou, Zongren Zhao, Da Tan, Chenhao Fang, Zhaoli Shen, Shun Li, Xianzhen Chen

**Affiliations:** 1 Department of Neurosurgery, Shanghai Tenth People’s Hospital, School of Medicine, Tongji University, Shanghai, China; 2 Department of Neurology, Ulm University, Ulm, Germany; 3 Department of Neurology, University of Pittsburgh School of Medicine, Pittsburgh, PA, USA

**Keywords:** artificial intelligence, biomarkers, gene expression regulation, genomics, metabolomics, nerve regeneration, neural pathways, proteomics, single-cell analysis, transcriptomics

## Abstract

Recently, with the rapid advancement of multi-omics technologies, including genomics, transcriptomics, proteomics, and metabolomics, new tools and approaches have been introduced for studying nerve injury and regeneration. This review highlights the application and progress of multi-omics in uncovering the mechanisms of nerve injury, guiding the development of regenerative strategies, and promoting clinical translation. By integrating multi-omics datasets, researchers can comprehensively track dynamic molecular changes following nerve injury, including abnormal gene expression, disrupted protein signaling, altered metabolic programs, and shifts in the immune microenvironment. Single-cell multi-omics technologies resolve cellular heterogeneity, revealing the distinct functions of neurons, glial cells, and immune cell subpopulations during the injury response. Spatially resolved transcriptomics maintain the spatial context of lesion and regeneration sites, enabling precise localization for targeted interventions. Multi-omics technologies not only identify key molecular players involved in nerve regeneration but also create opportunities for personalized medicine. Nonetheless, integrating multi-omics data poses technical challenges, including high dimensionality, batch effects, and algorithmic constraints, while ethical concerns related to stem cell therapy and gene editing require stringent oversight. To transition from structural reconstruction to functional remodeling, future research should emphasize artificial intelligence–driven data integration, organ-on-a-chip modeling, and cross-disciplinary collaboration to overcome existing technical barriers and accelerate the clinical application of neuroregenerative therapies.

**Facts**

• The combined analysis of single-cell spatial multi-omics has, for the first time, created a temporal and spatial dynamic map of the “neuron-glia-immune” triad interaction at single-cell resolution following neural injury, providing molecular coordinates for precise intervention.

• An artificial intelligence-driven cross-omics integration model (Transformer + Graph Neural Network) has established a closed loop that connects molecular features to individualized efficacy prediction.

• At the clinical translation level, a non-invasive efficacy assessment system has been established and recognized by the FDA as a breakthrough therapy, advancing neural regeneration into the “real-time parameter adjustment” era.

**Open questions**

• How can we simultaneously correct for technical batch effects and clinical heterogeneity (such as injury mechanisms and disease stages) in multi-omics data across different centers and technological platforms, while avoiding “over-correction” that leads to the loss of biological signals?

• When single-cell multi-omics reveal “cell state drift” and “microenvironment metabolic reprogramming” in neural regeneration, how can we design intervention windows with both temporal and spatial resolution to synergistically activate these dynamic processes rather than antagonize them?

• As clinical advancements in brain-machine interfaces, gene editing, and stem cell combination technologies progress, how can we establish a dynamic and updatable ethical governance framework that safeguards patient autonomy and data privacy while enabling rapid technological iteration?

## Introduction

Nerve injury is one of the primary causes of various diseases and can greatly impact patients’ quality of life (Lin et al., 2024). In recent years, the continuous advancement of multi-omics technologies has significantly broadened the research perspective in neuroscience (Wang et al., 2025b, d). Multi-omics encompasses genomics, transcriptomics, proteomics, metabolomics, and other omics disciplines (Liang et al., 2022). By integrating biological information from different levels, researchers can more comprehensively analyze the molecular mechanisms involved in nerve injury and repair, offering new insights into the pathogenesis and treatment of nerve injuries while deepening our understanding of neurological conditions (Zhang et al., 2024a, b). Despite a growing understanding of the cellular and molecular mechanisms underlying traumatic brain injury and spinal cord injury, there remains no effective cure (Lai et al., 2025). However, the application of multi-omics, combined with novel bioinformatics tools and advanced imaging techniques, is unveiling numerous new cellular and molecular targets that can be modulated, either individually or in combination, to promote precise repair of the damaged central nervous system (CNS) (Tedeschi et al., 2019). Moreover, the role of the immune system in peripheral nerve regeneration has gained increasing attention, with studies showing that eosinophils directly contribute to nerve regeneration by secreting cytokines that support this process (Pan et al., 2022; Liebendorfer et al., 2023).

Multi-omics is a systematic approach that analyzes biological complexity by integrating various layers of biological data, such as the genome, transcriptome, proteome, and metabolome (Wu et al., 2025). Its core advantage lies in overcoming the limitations of traditional single-omics methods by revealing dynamic molecular interaction networks through data integration (Pan et al., 2024). Depending on the research focus, multi-omics technologies can be categorized as follows: Genomics analyzes the effects of DNA sequence variations (e.g., single nucleotide polymorphisms and copy number variations) on disease susceptibility. Epigenomics examines epigenetic regulatory mechanisms, such as DNA methylation and histone modifications (Zhang et al., 2025a, b). Transcriptomics explores gene expression dynamics via RNA sequencing technologies (e.g., single-cell RNA-seq) (Zhang et al., 2025a, b). Proteomics investigates protein expression, post-translational modifications, and interaction networks using mass spectrometry. Metabolomics focuses on detecting small-molecule metabolites to assess cellular metabolic status and identify pathway abnormalities (Wang et al., 2023a, b). Additionally, radiomics combines medical imaging with artificial intelligence (AI) to quantitatively analyze tissue heterogeneity (Han et al., 2025a, b).

Multi-omics technologies are not only applied to nerve injury and repair but also offer significant advantages in the study of various neurological diseases (Wu et al., 2025). In neurodegenerative diseases, genomic and proteomic analyses have revealed the synergistic effects of amyloid-beta (Aβ) deposition and Tau protein phosphorylation, while metabolomics has uncovered the relationship between brain metabolic disorders and cognitive decline (Wu et al., 2025). In neurodevelopmental disorders such as autism spectrum disorders, multi-omics analyses have identified abnormal expression of genes related to synaptic function and epigenetic regulation (Osama et al., 2025). In neuroimmune diseases such as multiple sclerosis, single-cell transcriptomics has elucidated the interaction network between oligodendrocytes and immune cells, providing a foundation for targeted immune modulation (Smajić et al., 2022). The introduction of multi-omics technologies, particularly single-cell omics, not only enhances our understanding of cellular responses following nerve injury but also highlights the complex cellular interactions that occur during the nerve regeneration process.

However, despite the great potential of multi-omics technologies in nerve injury and regeneration research, several challenges remain. One key issue is the effective integration of multi-omics data and their translation into clinical applications. Most current studies focus on individual omics layers, while comprehensive, systematic investigations and methodological frameworks for multi-omics data integration and analysis are still insufficient (Qian et al., 2021; T et al., 2024). Therefore, future research should continue to explore and enhance the application of multi-omics technologies in nerve injury and regeneration, with the goal of developing more effective clinical treatment strategies and rehabilitation programs.

In summary, multi-omics technologies provide a novel platform and perspective for studying nerve injury and repair, significantly advancing our understanding of neurobiology. This paper aims to clarify the core concepts and classifications of multi-omics technologies, explore their applications in neurological diseases, and forecast future developments with an emphasis on interdisciplinary collaboration. It presents a comprehensive review of recent advances in multi-omics research related to nerve injury and regeneration, highlighting the unique strengths of these technologies in elucidating the molecular basis of neural damage. The paper also discusses cutting-edge applications of single-cell and spatial multi-omics in nerve regeneration, addresses the current challenges in multimodal data integration, and proposes practical solutions. Furthermore, it examines the emergence of artificial intelligence (AI)-driven precision diagnostic and therapeutic strategies, highlighting an evolving research paradigm characterized by “mechanism exploration – technological innovation – clinical application.” In light of ongoing challenges, the review proposes a forward-looking perspective: integrating multidimensional data to uncover the spatiotemporal specificity of nerve regeneration, with the ultimate goal of facilitating the clinical translation of laboratory discoveries.

## Search Strategy

**Figure 1:**
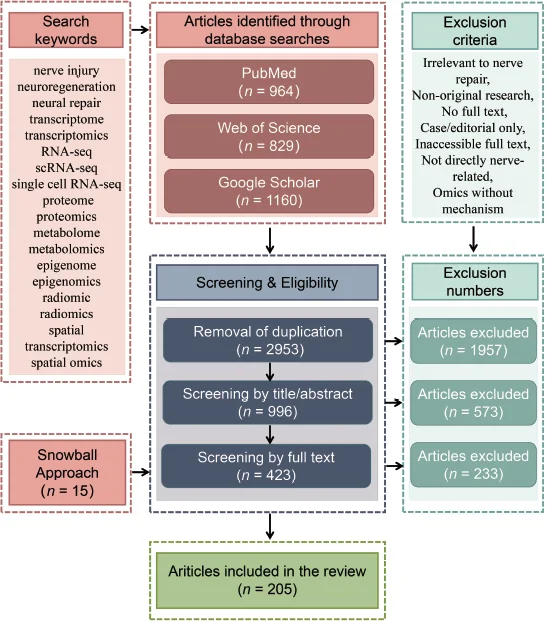
Search strategy and screening process for this review.

We conducted a comprehensive literature search spanning from 2000 to 2025, focusing predominantly on publications from 2014 to 2025, using three primary bibliographic databases: PubMed, Web of Science Core Collection, and Google Scholar. The search utilized the following keywords: “nerve injury,” “neuroregeneration,” “neural repair,” “transcriptome,” “transcriptomics,” “RNA-seq,” “single-cell RNA sequencing (scRNA-seq),” “proteome,” “proteomics,” “metabolome,” “metabolomics,” “epigenome,” “epigenomics,” “radiomic,” “radiomics,” “spatial transcriptomics,” and “spatial omics.” Two independent reviewers screened titles and abstracts to identify studies relevant to multi-omics investigations of neural injury or repair. Discrepancies and uncertain cases were resolved through adjudication by a third reviewer. Studies were excluded at this stage if they (1) did not focus on nerve injury or regeneration, (2) were not original research, or (3) were not available in full text. A full-text assessment was then conducted on the remaining 423 articles using predefined inclusion and exclusion criteria, which eliminated (1) case reports, editorials, and meeting abstracts without full text, (2) inaccessible full texts, (3) studies not directly addressing the mechanisms or functions of nerve repair and regeneration, and (4) papers in which multi-omics analysis was only used as a reference to biosignatures without in-depth mechanistic analysis. Additionally, six relevant studies were identified through snowballing, and 205 studies were included for qualitative synthesis (**Figure 1**).

## Overview of Multi-Omics Technologies for Nerve Injury and Regeneration

### Overview of multi-omics technologies

**Figure 2:**
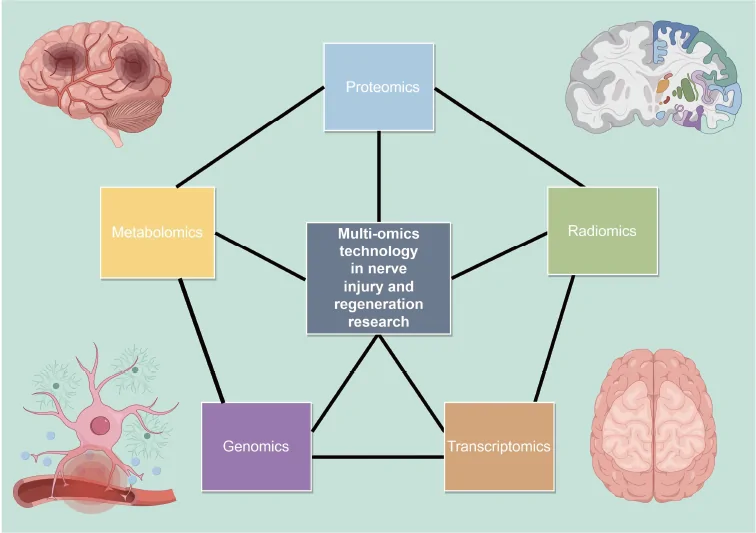
Multi-omics technologies in the research of nerve injury and regeneration.

Multi-omics technologies aim to comprehensively capture the complexity of biological systems by seamlessly integrating data across multiple levels, including genomics, transcriptomics, proteomics, and metabolomics. This approach offers a novel platform and perspective for investigating underlying mechanisms and guiding therapeutic strategies (Wu et al., 2025; **Figure 2**).

### Genomics

The application of genomics in nerve injury research has primarily focused on identifying genes and genetic variants associated with nerve injury and regeneration (Currais et al., 2025). Through genome-wide association studies (GWAS), researchers can pinpoint genetic markers linked to recovery outcomes, which may influence an individual’s response to injury and regenerative capacity (Zhuo et al., 2025). For instance, mutations in specific genes may increase the sensitivity of nerve cells to damage, thereby impairing their ability to regenerate (Huang et al., 2019). Furthermore, comparative genomic analyses across different species can uncover evolutionary mechanisms underlying nerve regeneration, offering novel targets for future clinical therapies. CRISPR, a powerful tool in functional genomics, provides new opportunities to unravel the mechanisms of neurological diseases and identify therapeutic targets (Kampmann, 2020). By enabling genome editing and precise control of gene expression in experimental disease models, CRISPR allows researchers to assess the functional consequences of genetic alterations in human cells. When combined with induced pluripotent stem cell technology, patient-derived cells can be differentiated into various neural cell types or brain organoids, effectively mimicking disease-associated gene expression changes and helping to identify causal molecular events (Jones et al., 2022; Leng et al., 2022). Additionally, genome engineering tools such as transcription activator-like effectors, along with CRISPR and related systems, provide innovative methods for precisely modifying genome sequences and gene expression levels (Lee et al., 2016). These advances are accelerating both basic research and clinical translation in neuroscience.

### Transcriptomics

Transcriptomics investigates gene expression in cells under specific conditions, providing insights into cellular responses following nerve injury and their dynamic changes during the regeneration process (Dai et al., 2025). Utilizing high-throughput RNA sequencing technologies, researchers can analyze transcriptomic profiles across different time points, allowing for the identification of genes that are upregulated or downregulated after nerve injury. These differentially expressed genes may play critical roles in regulating and promoting nerve regeneration (Yu et al., 2025; Ren et al., 2025).

Following nerve injury, neuronal damage signaling pathways trigger widespread transcriptional changes, and bioinformatics approaches have enabled the identification of numerous regeneration-related genes regulated by nerve injury (Ren et al., 2025). However, the mechanisms by which global transcriptomic changes are coordinated remain poorly understood. Epigenetic regulation has been shown to play a critical role in differentiating the robust axonal regeneration observed in the peripheral nervous system from the limited regenerative capacity of the CNS (Shin et al., 2017). In parallel, transcriptomic analyses have uncovered novel molecular mechanisms that promote nerve repair. By profiling the transcriptome of injured nervous tissue, researchers can map regulatory networks and identify potential therapeutic targets for enhancing regeneration. Overall, transcriptomic studies have significantly advanced our understanding of the molecular basis of nerve repair and have provided valuable insights for the development of targeted therapeutic strategies (Dulin et al., 2015; Shin et al., 2017).

Transcriptomics has been widely applied in neuroscience, with recent advances in single-cell transcriptomics and the development of cell type-specific and developmental stage-specific expression libraries significantly enhancing its utility in studying the CNS (Munro et al., 2009). However, extracting biologically meaningful insights from large-scale transcriptomic datasets remains a major challenge. Nonetheless, a current study has laid a solid foundation for understanding the molecular mechanisms underlying nerve injury and regeneration by analyzing gene expression changes through transcriptomic approaches (Qian et al., 2023).

### Proteomics

Proteomics, the study of the expression, function, and interactions of the entire protein complement within a cell, offers critical insights into nerve injury and regeneration (Claridge et al., 2021). By comprehensively analyzing protein content in cells or tissues, proteomics provides valuable data for understanding cellular responses to nerve injury. For instance, proteomic analyses have revealed distinct protein expression patterns at various stages and locations following peripheral nerve injury (PNI), shedding light on key proteins and signaling pathways involved in the regenerative process (Majima et al., 2025). Such alterations can influence neuronal survival and regenerative capacity. For example, after sciatic nerve transection in rats, elevated expression of acidic fibroblast growth factor and growth-associated protein 43 (GAP-43) was observed in the proximal stump at 10 days post-injury, while increased levels of protease nexin-1, ribosomal protein L9, and prosaposin were detected in the distal stump at 5 days, all of which are associated with nerve repair.

In-depth proteomic analysis thus enhances our understanding of the molecular basis of nerve regeneration and informs the development of novel therapeutic strategies.

Proteomic analysis can also detect alterations in serum protein profiles following nerve injury, offering insights into the systemic effects of such injuries on overall physiological homeostasis (Bolat et al., 2025). Research indicates that many of the differentially expressed proteins in the serum of mice with sciatic nerve injury are linked to inflammatory processes. Notably, serum from injured mice has been shown to induce pain hypersensitivity in otherwise healthy mice, implicating systemic inflammation in the pathogenesis of neuropathic pain (Zhou et al., 2023; Kanhema et al., 2025). Beyond peripheral injuries, proteomic investigations of brain and optic nerve injuries have further facilitated the identification of potential biomarkers and therapeutic targets, broadening the scope for effective clinical interventions in nerve injury management (Khan et al., 2025; Li et al., 2025a, b, c).

### Metabolomics

Metabolomics is a cutting-edge discipline focused on the comprehensive analysis of endogenous small-molecule metabolites, providing deep insights into the dynamic biochemical processes within living organisms (Kell, 2004). As highly sensitive indicators, metabolites reflect the internal metabolic environment and physiological states of cells. In the context of nerve injury and repair, metabolomics enables researchers to decode metabolic signal variations that occur post-injury, raising critical questions such as: How do these metabolic shifts influence cellular regeneration? Could specific changes in metabolite levels offer detailed insights into neuronal damage and subsequently modulate repair responses? While direct causal evidence is still lacking, advanced analytical techniques, such as gas chromatography-mass spectrometry and nuclear magnetic resonance spectroscopy, provide a robust platform for identifying potential biomarkers and guiding early diagnosis and intervention (Xu et al., 2025). Integrative metabolomics applications have proven especially valuable in revealing the biological regulatory mechanisms underlying peripheral nerve damage and regeneration. These approaches help illuminate how varying pathological conditions result in distinct metabolic phenotypes during post-injury recovery phases, thereby offering novel therapeutic directions and potential clinical benefits.

### Radiomics

Although radiomics is not a conventional molecular-level omics discipline such as genomics, transcriptomics, proteomics, or metabolomics, it has emerged as a powerful and complementary high-dimensional data modality in the study of neural injury and regeneration. While conventional imaging technologies primarily rely on morphological observations, radiomics introduces a transformative approach by quantitatively evaluating the heterogeneity of nerve injury through the analysis of texture features, metabolic distributions, and functional connectivity patterns in magnetic resonance imaging (MRI) or CT scans (Ma et al., 2025; Mekki et al., 2025). This shift toward quantitative imaging offers a novel strategy for assessing nerve injury (Song et al., 2025). Magnetic resonance neurography (MRN), in particular, enables precise visualization of peripheral nerve damage and the subsequent reinnervation process, which is critical for early diagnosis and therapeutic planning. A prospective multicenter study involving 60 patients with peripheral nerve injury is currently planned to assess the diagnostic and follow-up capabilities of MRN in comparison with conventional diagnostic tools (Aman et al., 2022). The outcomes of this study may provide not only a more accurate and timely method for diagnosis and treatment decisions but also a robust and precise means of monitoring nerve regeneration. Three-dimensional optical projection tomography has emerged as a valuable technique for visualizing nerve microstructure and injection-induced injury. A previous study on piggyback nerves has demonstrated its capability to reveal three-dimensional differences in the internal architecture of the median and lingual nerves, as well as the disruption and displacement of nerve bundles caused by injection injury, thereby offering a novel approach for investigating nerve microstructure and injury tolerance (Cvetko et al., 2015). Additionally, functional MRI (fMRI) provides a promising method for the early evaluation of nerve regeneration following injury repair. Experimental studies in rat brains have shown that fMRI can detect alterations in neuronal network connectivity after nerve injury and subsequent repair, offering a powerful clinical tool for monitoring therapeutic outcomes and guiding treatment strategies (Li et al., 2014; Grandjean et al., 2023).

## Multidimensional Analysis of Mechanisms of Nerve Injury

### Target selection and mechanism elucidation

Currently, the mechanism underlying nerve injury is not only complex but also highly multifaceted, involving numerous intricate biochemical reactions and multiple sub-processes within each reaction (Chun et al., 2022). Throughout the course of nerve damage, mechanisms such as cell death, inflammatory responses, and cellular regeneration become deeply intertwined. With the rapid advancement of multi-omics technologies, scientists are now able to investigate these intricate mechanisms from multiple dimensions through interdisciplinary and cross-field approaches, thereby enhancing our understanding of disease progression and identifying potential therapeutic targets (Zhou et al., 2025a, b). For instance, integrated analyses combining transcriptomics and metabolomics enable detailed exploration of complex cellular pathways, not only revealing dynamic gene expression patterns but also uncovering hidden intracellular regulatory mechanisms triggered by nerve injury (Zhou et al., 2025a, b). These insights open new avenues for treatment strategies and assessing therapeutic efficacy. Notably, significant alterations in metabolic fluxes, particularly in purine and amino acid pathways, have been observed across various tissues of the nervous system following neuralgia. These changes have a direct effect on intracellular energy metabolism and oxidative stress levels, ultimately influencing the capacity for nerve repair (Zeng et al., 2022; Kang et al., 2025). In terms of target selection, integrated multi-omics analyses have highlighted key transcription factors and signaling pathways associated with nerve regeneration. For example, transcription factors ATF7IP and JUNB have been shown to play critical roles in determining cell fate, with their altered expression closely linked to neural cell regeneration (Missinato et al., 2023). These findings offer promising directions for precision medicine, suggesting that targeted therapies focusing on such molecular pathways may significantly enhance treatment outcomes for nerve injury.

### Multiomics integration and biomarker discovery

**Figure 3:**
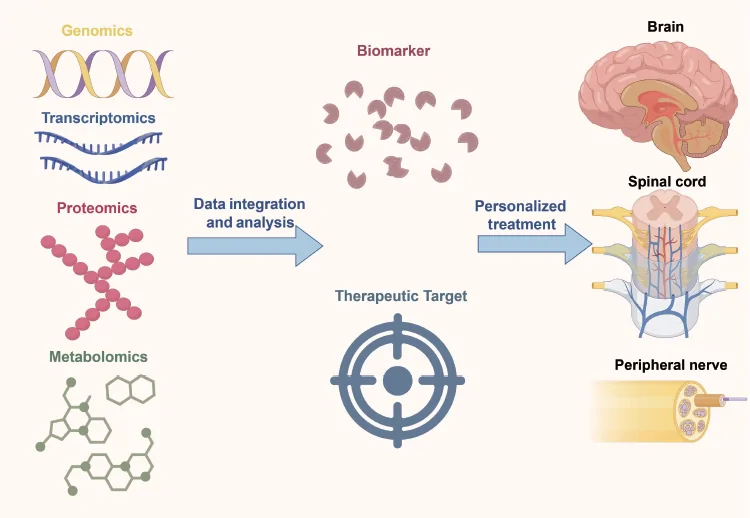
Multi-omics technologies promote biomarker discovery.

Combining multiple omics is a significant advancement in the discovery of biomarkers for nerve injury, fundamentally transforming research approaches within the neurosciences (**Additional Table 1**). By integrating diverse omics layers and constructing comprehensive multidimensional maps of nerve injury that incorporate complex molecular biology, researchers can identify specific biomarkers closely associated with both the injury process and subsequent regeneration (**Figure 3**). For instance, in a well-designed spinal cord injury model, the integration of transcriptomic and metabolomic analyses revealed numerous differentially expressed genes and metabolites, which were found to play pivotal roles in the recovery process following nerve injury. These molecular signatures have emerged as key candidates for further investigation (Ou et al., 2024). Furthermore, in a compelling multi-omics study on cerebral ischemia-reperfusion injury, researchers demonstrated that variations in metabolites and microbial communities were strongly associated with treatment outcomes (Shi et al., 2024), thereby broadening the scope for future interventions. Collectively, the in-depth investigation of these biomarkers has laid a solid foundation for earlier diagnosis and the development of personalized treatment strategies, contributing to more targeted and precise clinical therapies for nerve injuries.

**Additional Table 1:** Multiomics studies identifying biomarkers of nervous system injury and repair published from 2020-2025

Model/Context	Multiomics methods	Biomarker/Key finding	Reference
Human & mouse SCI	Proteomics + transcriptomics + metabolomics	Complement & lipid markers after exercise	Zhou et al., 2025b
Rat SCI and bladder injury	scRNAseq + spatial transcriptomics	DNAdamage & inosinesensitive markers	Wang et al., 2025d
Mouse ischemic brain	scRNAseq + spatial transcriptomics	Lgals9 as a potential treatment target	Han et al., 2024
Mouse SCI (various severities & ages)	snRNA-seq + transcriptome + epigenome + spatial transcriptomics	Pre-primed neuronal populations; stress vs reorganization tradeoff; Tripartite barrier re-establishment required; gene therapy restored walking in old mice;	Skinnider et al., 2024
Rat SCI + ES	Transcriptomics + proteomics	Potential therapeutic targets and pathways for SCI recovery	Li et al., 2024
Human AD tissue	scRNA-seq + spatial transcriptomics	NGFR promotes neurogenesis by inhibiting astroglia Lcn2/Slc22a17 signaling	Siddiqui et al., 2023
Mouse SCI	scRNAseq + spatial transcriptomics	Spatiotemporal microglial repair markers	Brennan et al., 2022
Rat SCI	Transcriptomics + metabolomics	Purine metabolism disruption	Pang et al., 2022
Rat PNI	snRNAseq + snATAC-seq	Proregenerative singlecell atlas	Lovatt et al., 2022
Human AD	Transcriptomics + proteomics	BIN1 restrains microglial proinflammatory activation and DAM phenotype	Sudwarts et al., 2022
Rat SCI	Proteomics + transcriptomics	Immune/lipid ism proteins	Yao et al., 2021
Human AD	Proteomics + transcriptomics	Identified conserved glial/inflammatory & synaptic proteomic networks; potential biomarkers GFAP, C1q, YKL-40	Swarup et al., 2020
Rat stroke	Proteomics + transcriptomics + metabolomics	MANF elevated in neurons & glia; protective exogenous MANF	Teppo et al., 2020
iPSCderived neurons & mouse AD	Proteomics + transcriptomics + metabolomics	Intracellular Aβ induces cell death resembling oxytosis/ferroptosis, which can be rescued by ferroptosis inhibitors	Huang et al., 2020

Each study included in Table 1 used at least two multi-omics approaches to experimentally investigate biomarkers associated with nervous system injury and repair. AD: Alzheimer's disease; Aβ: amyloid-beta; C1q: complement component 1q; DAM: disease-associated microglia; ES: electrical stimulation; GFAP: glial fibrillary acidic protein; MANF: mesencephalic astrocyte-derived neurotrophic factor; GFR: nerve growth factor receptor; PNI: peripheral nerve injury; SCI: spinal cord injury; scRNA-seq: single-cell RNA sequencing; YKL-40: chitinase 3-like protein 1.

These findings offer significant insights into the study of nerve injury and regeneration. First, the application of multi-omics technologies has effectively addressed the limitations of traditional single-layer analyses by enabling the construction of comprehensive molecular maps that integrate gene expression, protein interactions, and metabolic pathways (Wang et al., 2021). This advancement has revealed the dynamic coupling mechanisms within molecular networks following nerve injury and laid the foundation for identifying temporally specific biomarker systems with synergistic biological functions. Second, the integration of cross-omics data with tissue microenvironment analysis has established a multi-level research framework that encompasses molecular profiles, cellular phenotypes, and tissue remodeling processes (Wang et al., 2023a, b; Hu et al., 2023). For example, studies on spinal cord injury have uncovered a gene–metabolite regulatory axis that not only clarifies the biochemical basis of neuroplasticity but also pinpoints critical therapeutic targets involved in transmembrane signal transduction (Hu et al., 2023). Furthermore, the combination of spatial genomics with single-cell technologies has significantly enhanced the precision of injury microenvironment analysis. By mapping the spatial co-localization of lipid metabolic gradients and regenerative gene expression, researchers have developed novel therapeutic strategies targeting microenvironment-specific compartments, signaling a shift from broad-based treatments to finely targeted interventions (Yang et al., 2025; Tan et al., 2025). Finally, the emergence of AI-driven, real-time multi-omics platforms marks a transformative step in neuroregenerative medicine. These platforms promise to usher in a new era characterized by dynamic monitoring, intelligent prediction, and precision intervention. Such innovations aim to transcend the limitations of empirical treatment by integrating individualized molecular signatures, microbiome interactions, and other multidimensional data. This comprehensive, personalized approach holds great promise for improving therapeutic outcomes in patients suffering from challenging conditions such as paralysis and neurodegenerative diseases (Maity et al., 2025; Osama et al., 2025).

## Facilitating Role of Multi-Omics Technology in Nerve Regeneration

### Signaling pathway analysis in regeneration process

The application of multi-omics technologies holds significant promise in advancing nerve regeneration research, particularly through the in-depth analysis of the signaling pathways that orchestrate this complex biological process. By integrating multiple layers of biological information, such as genomics, transcriptomics, proteomics, and other omics datasets, Researchers are now equipped to systematically elucidate the molecular mechanisms underlying nerve repair (**Additional Table 2**). Recent studies have highlighted the pivotal roles of specific signaling pathways, including Wnt, Notch, and TGF-β, in regulating the regenerative response following nerve injury (Fiddes et al., 2018; Tedeschi et al., 2019; Zhuo et al., 2024). Notably, the Wnt/β-catenin signaling pathway has been shown to be intimately involved in neuronal survival, proliferation, and differentiation, with its activation after injury markedly enhancing the regenerative process (Tedeschi et al., 2019). Similarly, the Notch signaling pathway plays a critical role in the maintenance and differentiation of neural stem cells, and its modulation can significantly influence the efficacy of nerve regeneration (Fiddes et al., 2018).

**Additional Table 2:** Key signaling pathways in neural regeneration and their multi-omics evidence

Signaling pathway	Functional role	Multiomics evidence	Key factor/gene	Representative reference
Wnt/β-catenin	Promote neural progenitor cell proliferation, differentiation, axon guidance and growth cone expansion	Transcriptomics, proteomics, and scRNA-seq	*β-Catenin, TCF4, Fzd*, and *Axin2*	Gao et al., 2021; He et al., 2024
Notch	Maintain stemness, prevent premature differentiation, and control lineage decisions	scATAC-seq, single-cell transcriptomics, and chromatin accessibility	*Notchl, Hes1, Dili*, and *Jagged1/2*	Fiddes et al., 2018
TGF-β/Smad	Inhibit inflammation, regulate scar formation and ECM remodeling, and promote neural repair gene expression	Proteomics, transcriptomics, and metabolomics	*TGF-β1/2/3, Smad2/3, Sox9*, and *CTGF*	Zhuo et al., 2024
MAPK/ERK	Mediate neurotrophin signaling, promote axonal regeneration and synaptic plasticity	Phosphoproteomics, proteomics, and metabolomics	*ERKl/2, MEK, Ras, c-Fos*, and *Elk-1*	Cervellini et al., 2018
PI3K/AKT/mTOR	Support neuronal survival, regulate metabolism, and resist oxidative stress and apoptosis	Transcriptomics, proteomics, and phosphoproteomics	*PI3K, AKT1/2, mTOR, PTEN*, and *GSK3*β	Xian et al., 2021
JAK/STAT	Regulate immune cell activity, inhibit glial scar formation, and promote astrocyte functional differentiation	Transcriptomics, proteomics, and scRNA-seq	*JAK1/2, STAT3*, *SOCS3*, and *IL-6*	Ma et al., 2023
Hippo/YAP	Balance cell proliferation and apoptosis, regulate polarity and architecture in repair	Epigenomics, transcriptomics, and chromatin accessibility	*YAP, TAZ, MST1/2, LATS1/2, TEAD*	Huang et al., 2019
Shh	Promote neural stem cell proliferation and migration, and regulate early neurodevelopment	Transcriptomics, and spatial transcriptomics	*Shh, Ptch1, Smo*, and *Gli1/2/3*	Hu et al., 2024
BMP/Smad	Dual role: inhibit axonal growth but promote glial differentiation and stabilization	Proteomics, metabolomics, and scRNA- seq	*BMP2/4/7, Smad1/5/8*, and *Id1/3*	Akram et al., 2022
FGF/FGFR	Enhance axonal growth and synaptogenesis, and promote repair	Transcriptomics and spatial transcriptomics	*FGF2, FGFR1, ERK*, and *CREB*	Li et al., 2025a
VEGF/VEGFR	Facilitate neurovascular coupling and angiogenesis during neural repair	Transcriptomics and proteomics	*VEGF-A, VEGFR2, NRP1*, and *ERK*	Brudno et al., 2013

The pathways summarized here are implicated in various aspects of neural repair, such as inflammation regulation, cell survival, stem/progenitor cell activation, axonal regrowth, and synaptic remodeling. BMP: Bone morphogenetic protein; CREB: cAMP response element-binding protein; CTGF: connective tissue growth factor; ECM: extracellular matrix; ERK: extracellular signal-regulated kinase; FGF: fibroblast growth factor; FGFR: fibroblast growth factor receptor; GSK3β: glycogen synthase kinase 3 beta; IL-6: interleukin-6; JAK: Janus kinase; LATS1/2: large tumor suppressor kinase 1/2; MEK: mitogen-activated protein kinase kinase; MST1/2: mammalian sterile 20-like kinase 1/2; mTOR: mechanistic target of rapamycin; NRP1: neuropilin-1; PI3K/AKT/mTOR: phosphoinositide 3-kinase/protein kinase B/mechanistic target of rapamycin; PTEN: phosphatase and tensin homolog; Smad: signaling molecules downstream of TGF-β receptors; STAT3: signal transducer and activator of transcription 3; VEGFR2: vascular endothelial growth factor receptor 2; YAP: Yes- associated protein.

In a carefully designed mouse model of PNI, transcriptomic analysis revealed a significant upregulation in the expression of several genes associated with post-injury regeneration compared with non-injured controls. These genes were primarily involved in key processes such as cell proliferation, migration, and differentiation, all of which are critical to effective nerve repair (Gong et al., 2019; Dai et al., 2023). Numerous studies using high-throughput sequencing have identified a range of candidate transcriptional regulatory molecules, including transcription factors and their target genes, that play essential roles during the regeneration phase (Cho et al., 2023; Wang et al., 2025b). It is reasonable to hypothesize that the dynamic changes in transcription factor expression following nerve injury contribute significantly to the regeneration process. Investigating these transcriptional regulators and their associated signaling pathways may offer deeper insight into the molecular mechanisms of nerve repair and potentially lead to the development of more effective therapeutic interventions for patients with nerve damage.

### Progress of therapeutic targets

Neural regeneration is the biological process through which the central and peripheral nervous systems repair damage via axonal regeneration, synaptic remodeling, and the restoration of cellular function after injury (Ferreira et al., 2012). However, the regenerative potential of the adult mammalian CNS remains extremely limited, primarily due to an inhibitory microenvironment characterized by molecules such as Nogo-A, MAG, and Omgp, as well as the inherently low regenerative capacity of mature neurons (Zheng et al., 2023). In contrast, the peripheral nervous system (PNS) exhibits a more favorable regenerative environment and stronger cellular responses. To better illustrate the fundamental differences in regenerative responses between the CNS and PNS, **Additional Table 3** summarizes key distinctions in regenerative capacity, inflammatory response, glial scarring, remyelination mechanisms, and neural stem/progenitor cell dynamics. In recent years, the rapid advancement of multi-omics technologies has offered novel insights into the molecular underpinnings of nerve regeneration (Liu et al., 2024). By integrating data from genomics, transcriptomics, proteomics, metabolomics, and epigenomics, researchers can systematically elucidate key regulatory networks and uncover novel therapeutic targets for neuroregeneration (Akram et al., 2022). GWAS and scRNA-seq have identified several key genes associated with nerve regeneration (Choi et al., 2025); notably, components of the Ras homolog gene family member/Rho-associated protein kinase (Rho/ROCK) signaling pathway, such as Ras homolog gene family member A (RhoA) and Rho-associated protein kinase I (ROCK)-I/II, have been shown to mediate axonal growth inhibition by molecules such as Nogo protein A (Nogo-A) and myelin-associated glycoprotein (MAG). Transcriptomic analyses reveal dynamic, time-dependent gene expression changes following nerve injury, including upregulation of pro-regenerative genes (e.g., GAP-43, STAT3) and downregulation of pro-inflammatory cytokines (e.g., interleukin-6 [IL-6], tumor necrosis factor-alpha [TNF-α]) following Rho/ROCK inhibition, thus providing a molecular rationale for targeted therapies (Lingor et al., 2008). Epigenomic approaches, such as histone modification and DNA methylation profiling, show that HDAC inhibitors can promote axonal regeneration by altering chromatin states (Gaub et al., 2011; Puttagunta et al., 2014). Proteomic methods, particularly mass spectrometry, have clarified phosphorylation-dependent regulatory mechanisms, such as CSPG-mediated phosphorylation of MLCP through ROCK-II activation, which can be counteracted by ROCK inhibitors (Kalinski et al., 2019; Cho et al., 2023). Additionally, protein interaction assays such as co-immunoprecipitation and yeast two-hybrid analysis have identified RhoA-CRMP4b binding as a viable target for competitive inhibition strategies (Hall et al., 2001). Proteomics also plays a vital role in evaluating the efficacy and specificity of pharmacological agents such as fasudil and Y-39983 by monitoring their impact on downstream effectors (Sagawa et al., 2007; Zhu et al., 2021). Meanwhile, metabolomics has highlighted the critical role of energy metabolism in nerve regeneration, revealing that increased ATP demand is supported by hypoxia-inducible factor 1-alpha-mediated activation of glycolysis, as seen in studies investigating CoCl₂ in conjunction with ROCK inhibition (Sathyamurthy et al., 2024). Furthermore, sphingolipid metabolites such as S1P, which are crucial for neuronal membrane stability, exhibit changes correlating with ROCK activity, suggesting that dual-targeting of metabolic and signaling pathways may enhance regenerative outcomes (Alecu et al., 2017).

**Additional Table 3:** Comparison of regenerative features between CNS and PNS after injury

Feature	CNS	PNS
Regenerative capacity	Severely limited; glial scar formation impedes axonal extension	Strong regenerative ability; activated Schwann cells support axonal regrowth
Inflammatory response	Chronic and sustained, predominantly mediated by microglia and astrocytes	Self-limiting inflammation; macrophages efficiently clear myelin and debris
Glial scar formation	Reactive astrocytes form a dense glial scar, producing physical and chemical barriers that inhibit regeneration	No apparent scar barrier impeding regeneration
Remyelination mechanism	Relies on oligodendrocyte lineage; remyelination is slow and often incomplete	Robust support from activated Schwann cells facilitates effective remyelination
Neural stem/progenitor cells	Limited niches (e.g., hippocampus and SVZ); low proliferative activity	Schwann cells can be reprogrammed or activated; neural progenitor-like cells present in adult PNS

This table provides a comparative overview of the major biological differences between CNS and PNS in terms of their response to injury and regenerative potential. CNS: Central nervous system; PNS: peripheral nervous system; SVZ: subventricular zone.

Multi-omics and bioinformatics are essential tools for accelerating the identification of therapeutic targets in neural regeneration research (Qian et al., 2023). By integrating genomic data, transcriptome sequencing, proteomic profiles, and other datasets through machine learning techniques, researchers can uncover complex regulatory network patterns that optimize our understanding of nerve repair. For instance, the Rho/ROCK pathway is cross-regulated by the PI3K-Akt and MAPK pathways, and studies have shown that NSAIDs can inhibit ROCK while simultaneously stimulating the PI3K-Akt pathway (Zhang et al., 2023; Beljkas et al., 2024). Multi-omics approaches also facilitate biomarker selection, such as serum- or cerebrospinal fluid-derived miRNAs and metabolites, to assess the efficacy of ROCK inhibitors and support patient stratification (Zhang et al., 2024a, b). Using structural genomics and proteomics data, along with molecular docking technologies, can improve the binding efficiency between ROCK and its targets, thereby enabling the identification of patient subgroups (e.g., RhoA-expressing individuals) and informing personalized therapeutic decisions. Multi-omics integration supports the development of co-targeting strategies, such as combining ROCK inhibitors with neurotrophic factors, to stimulate synergistic activation of the MAPK pathway, enhancing regenerative outcomes (Xie et al., 2023). This technology accelerates translational progress from bench to bedside and helps elucidate the multilayered molecular mechanisms underpinning nerve regeneration (Chauhan et al., 2021). Therefore, these findings not only advance our ability to promote nerve regeneration but also provide novel avenues for investigating refractory neurological disorders. This marks a pivotal transition in life sciences toward a new era characterized by “big data focused on mechanisms and clinically transferable insights” (Gomes et al., 2023). Looking ahead, it is essential to establish an integrated multi-omics analysis pipeline, develop robust algorithms capable of linking cross-scale datasets, and validate systems biology-based predictive models using cutting-edge tools such as organoid microarrays (Tedeschi et al., 2019). These steps will be critical in realizing the goal of precise and effective neural repair.

## Clinical Perspectives

### New opportunities for personalized therapy

With the remarkable advancement of multi-omics technologies, the field of nerve injury and repair is approaching an unprecedented milestone, enabling patient-specific treatments based on individual molecular profiles for the first time in human history (Chauhan et al., 2021). The integration of various omics layers, such as genomics, transcriptomics, proteomics, and metabolomics, allows clinicians to design more precise, personalized, and effective therapeutic interventions (Chen et al., 2016). Research has demonstrated that cytokines, such as interleukin-4 (IL-4), play a critical role in promoting nerve repair (Daines et al., 2021; Pan et al., 2022). By precisely modulating the concentration of these cytokines, researchers have shown that post-injury recovery can be significantly improved. Consequently, identifying and validating such biomarkers through comprehensive multi-omics analyses is essential for optimizing personalized treatment strategies. In clinical settings, the successful implementation of individualized therapies requires strong interdisciplinary collaboration among experts from diverse fields, including molecular biology, bioinformatics, neurology, and data science (Wang et al., 2025a, b, c, d, e; Chun et al., 2025). This collaborative approach enhances the collective understanding of complex neurological injuries and fosters the targeted development of novel therapies (Liu et al., 2025a, b). Moreover, interpreting large-scale multi-omics datasets can be greatly enhanced by incorporating AI and machine learning, which offer powerful tools to guide clinicians in rapidly identifying optimal treatment pathways for each patient and advancing the realization of fully personalized neuroregenerative care.

### Application of multi-omics technologies in clinical trials

**Figure 4:**
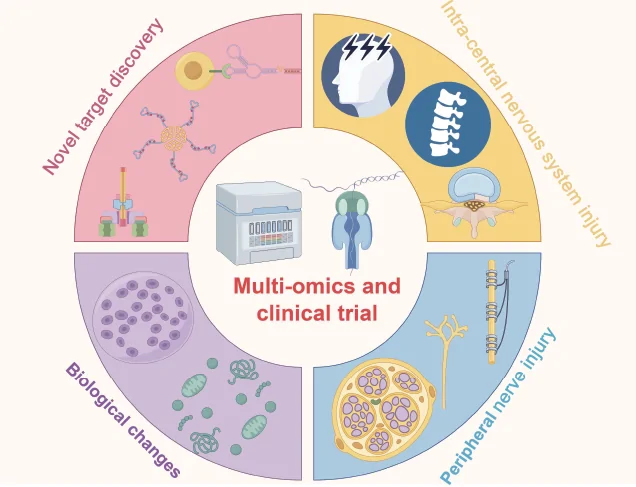
Considerable role of multi-omics technologies in clinical trials.

The integration of multi-omics technologies into clinical research is poised to transform the landscape of nerve injury and regeneration studies (**Figure 4**). By enabling a comprehensive analysis of the interactions between treatments and biomarkers, multi-omics approaches provide a more complete understanding of therapeutic efficacy and underlying biological mechanisms (Deri et al., 2025). During clinical trials involving PNI, the simultaneous collection and analysis of genomic, transcriptomic, and proteomic data allow researchers to identify critical molecular players involved in nerve regeneration, which may lead to the discovery of novel drug candidates (Chen et al., 2025a, b; Reinhold et al., 2025). Furthermore, the application of metabolomics offers insights into biological variations, such as metabolic shifts following nerve damage, providing clinicians with valuable information to guide treatment decisions dynamically. Monitoring these physiological changes in real time not only facilitates more accurate assessments of therapeutic outcomes but also enables timely interventions tailored to the evolving clinical context, thus optimizing the nerve repair process (Leno-Durán et al., 2024). Consequently, multi-omics technologies offer a new perspective and robust foundation for clinical studies of nerve injury and recovery, supporting the advancement of personalized medicine in this field. As multi-omics methodologies continue to evolve and gain broader adoption, their clinical applications are expected to expand, further accelerating translational efforts and enhancing patient outcomes.

### Progress and application prospects of multi-omics technologies in basic and clinical research

Multi-omics technologies offer a comprehensive perspective on biological systems by integrating data across various omics layers, including genomics, transcriptomics, proteomics, metabolomics, and microbiomics. In the context of nerve regeneration research, this approach provides several key advantages. First, it enables the identification of molecular networks involved in nerve injury and repair processes (Hu et al., 2023). Second, multi-omics facilitates longitudinal monitoring of disease progression and therapeutic responses, capturing the transition from acute injury to chronic repair phases and supporting the development of personalized treatment strategies through patient subpopulation stratification (Samanta et al., 2024). Third, it significantly enhances fundamental research in nerve regeneration (Pan et al., 2022). Fourth, it supports clinical translation by identifying therapeutic targets and biomarkers (Coroneos et al., 2025). Fifth, multi-omics expedites the development and validation of animal models used to study nerve regeneration (Magalhães et al., 2023). Sixth, it accelerates the discovery of novel molecular mechanisms and drug targets (Sagawa et al., 2007). Finally, it promotes the refinement of preclinical models for therapeutic testing (Choi et al., 2025). For example, the integration of CRISPR (clustered regularly interspaced short palindromic repeats) genome-wide screening with ATAC-seq (assay for transposase-accessible chromatin using sequencing) and RNA-seq (RNA sequencing) has revealed critical transcription factors such as ATF3 (activating transcription factor 3), ATF4 (activating transcription factor 4), C/EBPγ(CCAAT/enhancer-binding Protein Gamma), and CHOP (C/EBP homologous protein)/Ddit3 (DNA damage-inducible transcript 3), which modulate the balance between neuronal death and regeneration following axonal injury (Mantuano et al., 2011; Akram et al., 2022). This finding opens new avenues for the development of targeted neuroprotective therapies. Additionally, single-cell transcriptomics has been instrumental in mapping cellular heterogeneity within the injury microenvironment, uncovering astrocyte subtypes that secrete growth factors such as ciliary neurotrophic factor to support axonal regeneration, and highlighting the role of immune cell phenotypic shifts in the repair process. Moreover, multi-omics technologies facilitate disease molecular subtyping and target identification. For instance, in Alzheimer’s disease (AD), the integration of transcriptomic, epigenomic, and proteomic data has identified neuroprotective factors such as brain-derived neurotrophic factor and glial-derived neurotrophic factor and their regulatory networks. Copy number variations in the *HRH3* gene encoding the H3 receptor were found to contribute to disease pathology, providing a foundation for precision therapies (Nativio et al., 2020). Combining gut microbiome analysis with plasma and neurolipidomics has revealed that specific bacterial genera, including Lactobacillus and Anaerotruncus, influence neuroinflammation via modulation of sphingomyelin metabolism, thus informing metabolic intervention strategies (Yadav et al., 2014; Shi et al., 2025).

Multi-omics technologies play a crucial role in the clinical validation of biomarkers, extending beyond the identification of novel therapeutic targets to actively promoting personalized medicine through the analysis of a patient’s genomic profile and disease-specific characteristics (Tsai et al., 2015). It is important to highlight that dynamic metabolic tracking at single-cell resolution, as well as algorithms for integrating cross-scale data, from molecular to tissue to system levels, still require significant optimization (Luecken et al.,2019).

A major limitation in the field is the absence of standardized protocols for the collection, storage, and analysis of multi-omics data, which hinders the comparability and reproducibility of results across different research centers (Mangul et al., 2019; Cao et al., 2022). Moreover, ethical concerns surrounding the use of organoids, along with the increasing complexity of clinical trial designs, particularly the surge in sample size requirements due to the heterogeneity seen in conditions such as amyotrophic lateral sclerosis, highlight the urgent need for systematic and multidisciplinary solutions (Paganoni et al., 2020).

The application of multi-omics technologies in neural regeneration is continuously advancing, offering substantial value not only in elucidating the underlying mechanisms of neural repair but also in fostering the development of innovative therapeutic strategies (Wang et al., 2022). These evolving technologies are reshaping the landscape of neural regeneration research by enhancing the entire continuum of discovery, validation, and clinical translation. Their unique capacity to analyze complex disease mechanisms and design precise intervention strategies is steering neuroscience away from descriptive paradigms toward a mechanism-driven, functional medicine approach (Tian et al., 2022). With the rapid progress of spatial multi-omics and in situ metabolic imaging technologies, the future holds promise for real-time monitoring and closed-loop regulation of nerve regeneration, potentially achieving both structural and functional restoration of neurological damage (Wu et al., 2024). As technological advancements continue and interdisciplinary collaboration deepens, the application of multi-omics in addressing neurodegenerative diseases and traumatic neural injuries is expected to expand significantly.

## Latest Research Findings and Theoretical Framework

### Latest multi-omics research results

With the advancement of multi-omics technology, significant progress has been made in the field of nerve injury and regeneration research. Multi-omics analysis offers new insights into these complex biological processes by integrating large-scale data from various domains such as genomics, transcriptomics, proteomics, and metabolomics (Wu et al., 2025). This integrative approach allows for a deeper exploration of the intricate mechanisms underlying nerve injury and repair. For instance, numerous studies have clarified how cellular and molecular alterations following traumatic brain injury and spinal cord injury impact the structure and function of the nervous system (Nakagawa et al., 2025; Srivastava et al., 2025). Although a complete cure remains elusive, the innovative application of multi-layered omics continues to reveal novel therapeutic targets and guide the development of more effective treatment strategies.

Additionally, the investigation of low-intensity pulsed ultrasound has emerged as a promising, non-invasive approach to stimulate nerve regeneration (Peng et al., 2020). Its efficacy is attributed to the upregulation of neurotrophic factors and the activation of Schwann cells, which collectively enhance neural repair. Furthermore, inflammation, an inevitable component of nerve regeneration, plays a dual role; while mild inflammation promotes nerve healing, excessive inflammatory responses can exacerbate neural damage. Recent research has also focused on the use of mesenchymal stem cells (MSCs) in treating peripheral nerve injury (Aisaiti et al., 2024). MSCs possess remarkable regenerative capabilities and have demonstrated effectiveness in enhancing neural repair through various mechanisms, particularly in the context of nerve conduits. Embedding MSCs within these conduits, along with the controlled release of specific growth factors, has been shown to significantly influence the success of nerve regeneration (Liu et al., 2025a, b; Nevado-Sánchez et al., 2025).

### Integration and optimization of theoretical framework

**Figure 5:**
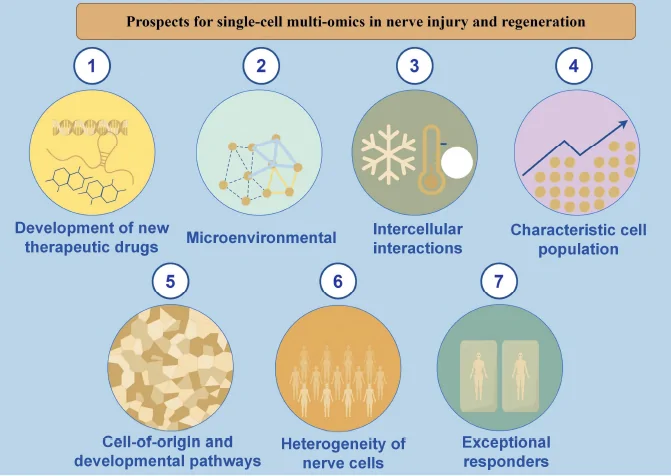
Prospects for the application of single-cell multi-omics in nerve injury and regeneration.

Conducting multi-omics studies on nerve injury and regeneration requires the continual refinement and integration of relevant theoretical frameworks to ensure sustained progress in the field. By leveraging the multi-omics data generated through these approaches, researchers can gain deeper insights into the biological processes underlying nerve damage and repair. The advent of single-cell multi-omics technologies has opened new avenues for understanding cellular heterogeneity at high resolution, offering a more nuanced perspective on the distinct roles individual cell types play during the complex processes of neural injury and regeneration (Kong et al., 2025; Gupta et al., 2025; **Figure 5**). Each cell type contributes uniquely to these processes, and deciphering how they coordinate remains an area of active investigation (Kanda et al., 2025).

Recent advancements in statistical and computational methods have greatly enhanced bioinformatics capabilities, enabling researchers to effectively integrate and analyze diverse multi-omics datasets. This integrative approach lays a robust theoretical foundation for translating findings into clinical practice (Li et al., 2025a, b, c; Srivastava et al., 2025). Consequently, it is essential to not only develop and refine analytical models but also to harmonize findings across multiple data modalities. A broader focus on discovering novel biomarkers involved in neural regeneration, rather than limiting investigations to a narrow set of targets, will be critical. Despite the promising potential, substantial work remains before multi-omics technologies can be routinely applied in individualized clinical settings for the diagnosis and treatment of nerve injury and repair (Hu et al., 2025; Han et al., 2025a, b).

In theoretical integration, priority should be given to multi-data frameworks within the context of multimodality, as reliance on single-omics data presents inherent limitations and challenges (Bersanelli et al., 2016). For instance, single-cell multi-omics technologies, such as scRNA-seq and scATAC-seq, have revealed diverse neuronal differentiation trajectories post-injury by simultaneously analyzing gene expression, chromatin accessibility, and RNA splicing (Luecken et al., 2019; Stuart et al., 2019). Moving forward, the development of algorithms that standardize cross-omics data fusion is essential. Deep learning–based mutual information consistency analysis offers one promising solution by addressing data heterogeneity, deciphering cellular relationships, and characterizing metabolic states within the microenvironment, particularly in applications involving spatial transcriptomics platforms like 10x Visium (Huang et al., 2017). Guided by systems biology, dynamic molecular network models should be constructed to simulate the spatiotemporal regulation of key signaling pathways, such as Wnt and Notch, during neural regeneration, thereby providing a theoretical foundation for precise therapeutic targeting (Ables et al., 2010; Nusse et al., 2017). Concurrently, AI-driven modeling approaches must be adopted to enhance predictive capabilities and intervention strategies by incorporating imaging and electrophysiological data (e.g., electroencephalography) into machine learning frameworks, including random forests and graph neural networks. This integrative approach enables the identification of biomarkers associated with regenerative responses and supports the prediction of therapeutic outcomes (Klepl et al., 2022; Shoeibi et al., 2022). For example, deep learning models can leverage single-cell epigenetic information to assess cell differentiation potential and inform organoid construction for transplantation, while structural equation modeling (SEM), a causal inference method, can isolate direct regulators within multi-omics datasets and minimize false-positive associations that may obscure biological interpretation (Li et al., 2020; Reed et al., 2021). Ultimately, successful clinical translation requires the formulation of precise, individualized treatment strategies, whereby regenerative targets identified through multi-omics are validated using CRISPR-Cas9 gene editing tools and delivered via transfection vectors such as adeno-associated virus or hydrogel nanoparticles, followed by functional assessment in organoid or humanized animal models (Komor et al., 2017; Tolabi et al., 2023).

In addition, there is an urgent need to establish dynamic biomarker monitoring systems, such as the lactate/pyruvate ratio and DNA methylation markers, in conjunction with adaptive clinical trial designs to enable the real-time optimization of therapeutic regimens (Timofeev et al., 2011). Concurrently, fundamental research must advance the understanding of cross-scale mechanisms to bridge the gap between molecular-level events and system-level functional outcomes. For instance, the integration of single-cell transcriptomics with spatial metabolomics can uncover the spatiotemporal associations between metabolic reprogramming (e.g., enhanced glycolysis) and the immune microenvironment, particularly in relation to M1/M2 macrophage polarization in spinal cord injury (Chen et al., 2025a, b). Additionally, the development of biomimetic platforms, such as organ-on-a-chip models, holds promise for simulating interactions among neural, immune, and vascular systems, while also elucidating the combined impact of mechanical stressors and biochemical signals on neural regeneration (Deng et al., 2023). Collectively, these advances will help transition nerve regeneration research from an empirical, observation-based approach toward mechanism-driven precision medicine, ultimately facilitating the efficient restoration of neural function.

### Artificial intelligence-driven: Multi-omics data integration and predictive modeling

**Figure 6:**
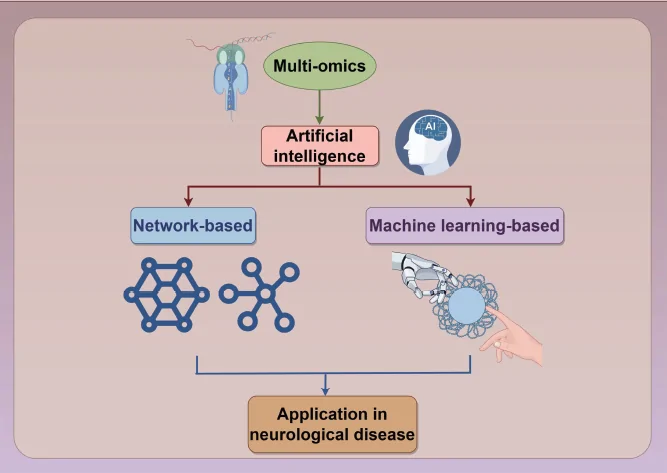
Artificial intelligence has brought revolutionary changes to the field of nerve damage and regeneration.

The in-depth application of AI in data integration and analysis is catalyzing a leapfrog advancement in multi-omics research, fundamentally transforming the study of neurological injury and regeneration (Gobira et al., 2025; **Figure 6**). The rapid accumulation of multidimensional genomic data has rendered traditional data processing approaches, predicated on statistical assumptions and basic correlation analyses, inadequate for managing the scale and complexity of high-dimensional, heterogeneous datasets. AI technologies, particularly advances in machine learning and deep learning algorithms, offer innovative solutions that are reshaping the multi-omics landscape. At the data integration level, AI enables seamless fusion of cross-scale, multi-omics information through sophisticated multimodal data fusion frameworks (Rames et al., 2025). For instance, in nerve injury research, AI algorithms can synchronize gene expression matrices from single-cell sequencing, proteomic profiles from mass spectrometry, radiomic features from high-resolution MRI, and clinical phenotypic data from electronic health records (Gobira et al., 2025). By using non-linear dimensionality reduction via autoencoders or constructing biomolecular interaction networks with graph neural networks, researchers can elucidate regulatory relationships across molecular layers and uncover cascading pathological mechanisms, such as epigenetic dysregulation leading to transcriptomic disorders and aberrant protein post-translational modifications (Hinton et al., 2006; Zitnik et al., 2018). This integrated analysis transcends the constraints of traditional single-omics studies and provides a comprehensive systems biology perspective for understanding the complex pathology underlying nerve injury.

In the realm of data analysis, deep learning models exhibit remarkable capabilities in feature extraction (Zhu et al., 2025). Convolutional neural networks excel at automatically detecting alterations in tissue microstructure through radiomic imaging data, while recurrent neural networks are particularly well-suited for capturing temporal dynamics within epigenomic datasets. Moreover, transformer architectures have demonstrated proficiency in modeling long-range dependencies involved in gene regulatory mechanisms. When applied to spinal cord injury research, these AI technologies have enabled the precise identification of critical regulatory nodes within large-scale gene expression profiles, thereby advancing our understanding of the complex molecular interactions underlying nerve regeneration.

At the clinical translation level, AI is establishing a comprehensive closed-loop system that seamlessly integrates basic research with clinical application (Andersson et al., 2025). Machine learning models leveraging multi-omics datasets enable the construction of individualized disease progression prediction systems (Trabilsy et al., 2025). Furthermore, reinforcement learning frameworks are being employed to optimize personalized treatment strategies by simulating interactions between various intervention modalities, such as the timing of stem cell transplantation, parameters of neuroelectric stimulation, and the patient’s unique multi-omics profile. This approach facilitates the recommendation of optimal therapeutic pathways, thereby advancing the realization of truly precise neuroregenerative medicine (Omar et al., 2025).

Looking ahead, generative adversarial networks are poised to significantly enhance the synthesis and augmentation of multi-omics datasets, effectively addressing the challenge of limited sample sizes in rare neurological diseases. Neural symbolic systems hold the promise of advancing from mere data pattern recognition to causal inference, thereby uncovering the fundamental biological principles underlying neural regeneration. Additionally, the development of brain-inspired chips is expected to dramatically improve the real-time processing capabilities and energy efficiency of multi-omics analyses, elevating performance by orders of magnitude (Woods et al., 2025). Collectively, these technological breakthroughs will establish a smart, integrated platform for nerve injury research, driving innovation in therapies aimed at neuroprotection, axonal regeneration, and neural circuit reconstruction, ultimately realizing the clinical goal of restoring functional recovery for more patients suffering from nerve injuries (Carlo et al., 2025).

## Controversial Points and Challenges in Nerve Injury and Regeneration Research

### Limitations of multi-omics techniques in nerve injury research

Despite significant advancements in multi-omics technology for nerve injury research, several critical limitations remain. For example, in studying pyrexia-induced neurological injury in the cerebral cortex, multi-omics analyses have illuminated relevant pathological mechanisms but still fall short of fully capturing the complex pathogenesis underlying neurological injuries (Zeiler et al., 2021). Similarly, while single-omics approaches face inherent challenges in fully elucidating spinal cord injury progression and guiding treatment, multi-omics methods, although promising, encounter practical difficulties, especially in data integration and analysis (Pan et al., 2025). In investigations of dystonia-like movements and metabolic disorders caused by peripheral nerve injuries, multi-omics can detect significant molecular alterations; however, deeper exploration of intricate mechanisms requires further technological enhancement and integration (Knorr et al., 2024). Moreover, multi-omics techniques traditionally struggle with capturing spatial information. For instance, in the study of traumatic spinal cord injury microenvironments, conventional methods often overlook crucial spatial details such as precise cell locations and cell-cell interactions, thereby limiting a comprehensive understanding of injury mechanisms (Wu et al., 2024). Thus, while multi-omics has driven considerable progress in neurological injury and regeneration research, multiple challenges remain that suggest important directions for future work. First, overcoming fragmented insights from single-omics perspectives necessitates the development of robust cross-omics data integration models, along with AI-driven causal inference algorithms to move beyond correlation towards mechanistic understanding (Rotroff et al., 2016; Chen et al., 2024). Constructing a time-series multi-omics database combined with mathematical modeling will enable prediction of optimal intervention time windows, while the integration of spatial genomics technologies will preserve critical cellular microenvironment localization information (Singhal et al., 2021). For example, combining spatial transcriptomics platforms such as 10x Genomics Visium (Mirzazadeh et al., 2023) with dynamic sampling strategies such as continuous biopsy (Devaux et al., 2016) can reconstruct detailed 3D spatiotemporal maps of nerve injury microenvironments, compensating for the limitations of traditional approaches that lose spatial and temporal resolution. Second, bridging the gap between functional validation and clinical translation demands the creation of CRISPR interference/activation libraries linked with organoid and animal model screening systems. The development of high-throughput phenotypic screening platforms and a translational workflow encompassing omics discovery, animal validation, organoid simulation, and clinical trials will accelerate application (Hu et al., 2021). Building this clinical translation bridge requires integrating diverse organoid models and establishing multimodal biomarker panels, nanomedicines targeting key regulatory nodes, and personalized rehabilitation strategies informed by metabolomic profiles of gut microbiota, thus enabling a leap from molecular mechanisms to precise therapeutic interventions (Weng et al., 2024; Italiano et al., 2025). Third, inherent technical constraints call for optimization of sample preprocessing, innovation in single-molecule assay development, and creation of advanced deep learning frameworks, such as graph neural networks, to analyze complex omics networks, along with the establishment of open, shared multi-histology knowledge graphs (Can et al., 2023). Finally, paradigm shifts in nerve regeneration research highlight the need to expand focus from simple “damage and repair” models to include microenvironmental regulation. Emphasizing extracellular matrix remodeling’s role in guiding axonal regeneration and supporting personalized treatment strategies via multi-histology typing will be critical for future progress.

### Technical challenges in data integration and multi-omics analysis

**Figure 7:**
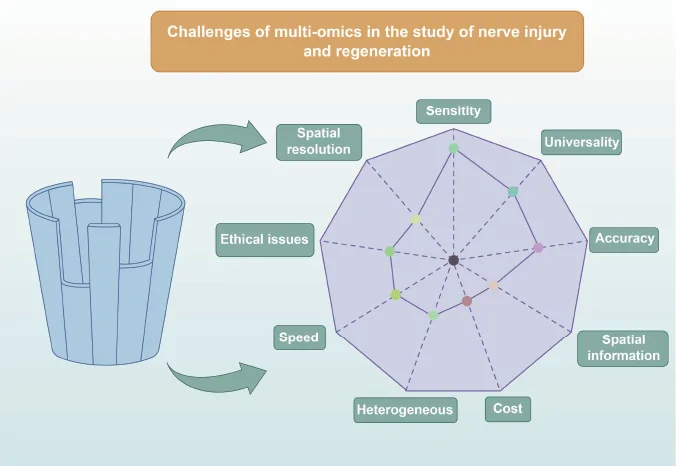
Challenges for multi-omics technologies.

The in-depth development of multi-omics research faces a series of complex technical bottlenecks spanning the entire process from data collection and integration to analysis and interpretation (Akabane et al., 2025; Kelliher et al., 2025). Multi-omics datasets are diverse, encompassing genomics, transcriptomics, proteomics, epigenomics, metabolomics, microbiomics, and more, each characterized by distinct data structures, measurement units, dynamic ranges, and noise profiles (**Figure 7**). For instance, genomics data typically consist of discrete sequence variants, transcriptomics data capture continuous gene expression levels, proteomics data exhibit high heterogeneity due to post-translational modifications, and metabolomics data display pronounced spatiotemporal dynamics (Mohammadi-Shemirani et al., 2023). This intrinsic heterogeneity causes a significant “dimensionality disaster” during data integration. Traditional linear normalization methods struggle to effectively remove systematic biases across different omics platforms, making batch effect correction a critical challenge that limits data comparability (Fan et al., 2024).

At the level of data analysis methods, existing technical systems struggle to address the complexity and high dimensionality inherent in multi-omics datasets (Zhang et al., 2025c). Traditional statistical approaches such as t-tests and ANOVA are insufficient for analyzing high-dimensional, incomplete data, particularly when missing value rates exceed 30% (Brady et al., 2015). In datasets comprising tens of thousands of features, dimensionality reduction techniques such as principal component analysis often fail to capture non-linear patterns, while correlation-based networks are susceptible to high false positive rates (Ringnér, 2008). Although machine learning algorithms such as support vector machines and random forests are capable of managing high-dimensional data, their limited interpretability restricts the extraction of meaningful biological insights. Deep learning models, including autoencoders, offer potential for advanced feature extraction but typically require large annotated datasets, resources that remain scarce in biological research contexts (Dahou et al., 2025). Moreover, data interpretation is further complicated by information overload, where critical molecular signals are obscured by background noise (Deshpande et al., 2023). To overcome these challenges, there is a pressing need for intelligent biomarker screening strategies that integrate pathway enrichment analysis, network topology metrics, and statistical models such as LASSO to enable the extraction of biologically actionable insights.

On the infrastructure side, multi-omics research produces massive volumes of data, whole genome sequencing alone can generate several terabytes, placing significant strain on existing storage and data retrieval systems. The absence of standardized data formats and the low reusability of data, often due to experimental variability, further impedes platform interoperability (Pan et al., 2025). Compounding these challenges are rising privacy concerns in clinical omics, as genetic data are inherently identifiable and sensitive. To address these issues, advanced privacy-preserving technologies such as homomorphic encryption and federated learning are urgently needed to facilitate secure, decentralized data sharing without compromising patient confidentiality.

## Limitations

Multi-omics technologies have been increasingly applied to the field of nerve injury and regeneration, opening new research avenues for scientists; this review article systematically summarizes existing results, but it also presents several limitations, not only in practical aspects such as technical methodologies, data integration, and clinical translation, but also in deeper dimensions including theoretical frameworks, ethical considerations, and interdisciplinary integration. While the paper highlights core omics domains such as genomics, transcriptomics, proteomics, and metabolomics, it lacks adequate coverage of emerging omics fields such as epitranscriptomics, lipidomics, and immunomics. The crucial roles of epigenetic modifications, such as DNA methylation and histone modifications, in neural regeneration are underexplored, and the potential value of spatial metabolomics in understanding the nerve injury microenvironment is not sufficiently addressed. Moreover, the review predominantly focuses on spinal cord injuries and peripheral nerve regeneration, with limited analysis of brain injuries (e.g., traumatic brain injury and ischemic stroke), despite their distinct pathophysiological mechanisms. Notably, the immune infiltration patterns following blood–brain barrier disruption differ markedly from those in peripheral nerves, yet the paper does not provide a comparative multi-omics analysis of the central versus peripheral nervous systems. Discussions around single-cell multi-omics technologies, such as CyTOF and spatial transcriptomics, remain confined to basic technical descriptions, lacking in-depth examination of their transformative potential on research paradigms. Although the review emphasizes the revolutionary promise of multi-omics in neuroregeneration, it also underscores its current shortcomings, which may serve as catalysts for further innovation. With the continued maturation of advanced technologies and increased interdisciplinary collaboration, multi-omics could evolve from a mere “toolbox” to a strategic “navigator,” propelling neuroregenerative medicine into a new era characterized by more precise diagnostics and effective interventions. However, the interpretations and applications discussed are still constrained by the limited maturity of multi-omics platforms, data quality, methodological designs, and the ongoing difficulty of translating findings from laboratory settings to clinical practice. Researchers must focus on enhancing technological standardization, building robust data-sharing infrastructures, and fostering cross-disciplinary partnerships to ensure smooth clinical translation. Additionally, current research faces two critical challenges: first, the complexity of batch effects arising from heterogeneous data sources (e.g., single-cell RNA-seq, radiomics, proteomics), each with distinct bias patterns; and second, the prevalent tendency to correct only for technical batch effects, while neglecting clinical heterogeneity, such as injury mechanisms and disease progression stages, that significantly impact biological interpretation. A recent publication, A Guide to ComBat Harmonization of Imaging Biomarkers in Multicenter, underscores the necessity of incorporating clinical covariates when correcting for batch effects in multicenter studies. Its insights are crucial for advancing multi-omics research in nerve injury and regeneration (Orlhac et al., 2022). Specifically, it reveals that differences in clinical feature distributions (e.g., injury type, age, and disease stage) across centers can obscure the distinction between biological signals and batch effects. Furthermore, it stresses that tissue- and disease-specific corrections are irreplaceable, as the nature of batch effects varies across biological contexts, such as inflammation-related genes in acute spinal cord injury being more susceptible to RNA-seq batch bias than in chronic phases, where different gene groups dominate, necessitating independent ComBat parameter estimation. Lastly, the use of covariates must align with the homogeneous batch effect hypothesis, meaning the validity of clinical grouping depends on the internal consistency of batch effect patterns within each subgroup, compelling neural researchers to rigorously validate the biological soundness of such classifications.

## Conclusions and Future Perspectives

**Figure 8:**
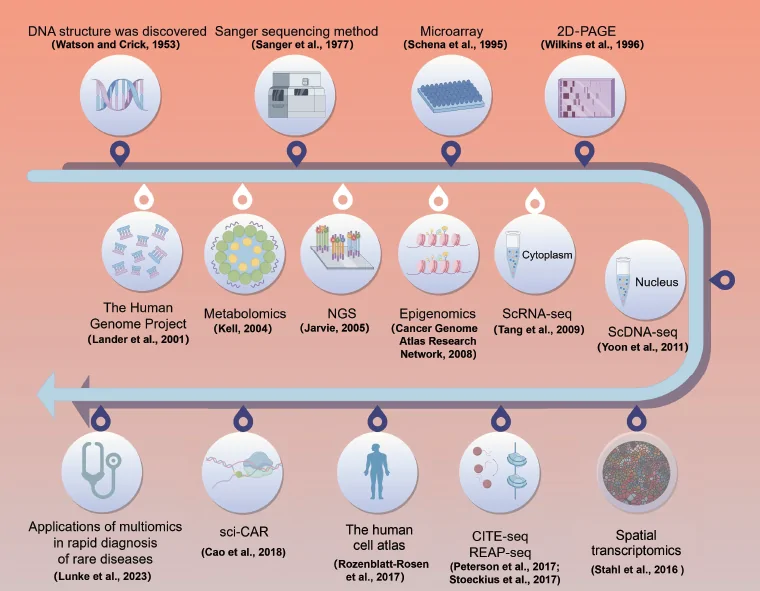
Milestones in the evolution of multi-omics technologies: from DNA discovery to precision medicine. The selected events are based on publicly available literature and landmark publications in the omics field. 2D-PAGE: Two-dimensional polyacrylamide gel electrophoresis; CITE-seq: cellular indexing of transcriptomes and epitopes by sequencing; epigenomics: epigenome-wide analysis; NGS: next-generation sequencing; REAP-seq: RNA expression and protein sequencing; ScDNA-seq: single-cell DNA sequencing; sci-CAR: single-cell combinatorial indexing chromatin accessibility and mRNA; ScRNA-seq: single-cell RNA sequencing.

Since Watson and Crick unveiled the DNA double helix in 1953 (Watson and Crick, 1953), omics technologies have advanced in transformative waves: Sanger sequencing in 1977 laid the foundation for high-precision genomics (Sanger et al., 1977); microarrays in 1995 (Schena et al., 1995) and 2D-PAGE in 1996 initiated high-throughput transcriptomics and proteomics (Wilkins et al., 1996); the Human Genome Project draft in 2001 (Lander et al., 2001) and the emergence of metabolomics in 2004 marked a shift toward comprehensive small-molecule profiling (Kell, 2004); next-generation sequencing in 2005 vastly accelerated data generation (Jarvie, 2005); from 2008 to 2012, initiatives such as ENCODE and TCGA integrated epigenomics and multi-omics to map complex regulatory networks (Cancer Genome Atlas Research Network, 2008; ENCODE Project Consortium, 2012); single-cell RNA-seq in 2009 (Tang et al., 2009) and single-cell DNA-seq in 2011 (Yoon et al., 2011) enabled the discovery of cellular heterogeneity beyond population-level data; spatial transcriptomics in 2016 (Ståhl et al., 2016) introduced spatial resolution to gene expression; and multimodal single-cell techniques, such as CITE-seq (Stoeckius et al., 2017), REAP-seq (Peterson et al., 2017), and sci-CAR (Cao et al., 2018), allowed simultaneous analysis of proteins, chromatin, and RNA within individual cells. The human cell atlas in 2017 (Rozenblatt-Rosen et al., 2017) had begun to establish a global reference map of human cell types, and by 2023, multi-omics technologies had begun enabling rapid molecular diagnoses for rare diseases (Lunke et al., 2023; **Figure 8**). This rapid evolution of multi-omics presents an unprecedented opportunity to investigate nerve injury and the complex processes of nerve regeneration. By exploring vast and interconnected layers of biological data, from the genome and transcriptome to the proteome and metabolome, multi-omics technologies offer profound insights into the molecular mechanisms underlying nerve injury, while also providing innovative perspectives for understanding regeneration.

Recent studies highlight that nerve injuries trigger dramatic biomarker fluctuations, and multi-omics approaches help uncover these key indicators, enhancing clinical diagnosis and enabling personalized treatments tailored to individual biological profiles. However, discrepancies often arise across multi-omics studies due to differences in sample sources, experimental setups, and data processing techniques, necessitating careful interpretation. Addressing this requires active participation in multicenter collaborations and rigorous cross-validation to reconcile divergent findings. Building expansive biomarker databases from diverse laboratories will create a more robust foundation for further discovery and clinical translation. The application of multi-omics in personalized medicine is a revolutionary leap for healthcare, enabling precise and individualized treatment strategies based on genomic, phenotypic, and other omic-layer data. For instance, transcriptomic profiling can predict patient-specific responses to drugs, minimizing adverse effects and enhancing treatment efficacy. Looking ahead, the integration of AI and big data analytics with multi-omics will streamline the implementation of personalized medicine in neurological injury and regenerative care, potentially leading to groundbreaking therapeutic advancements. The ongoing progression of multi-omics technologies deepens our understanding of nerve injury and recovery mechanisms, and we must embrace this momentum by venturing into unexplored research territories and ensuring the clinical relevance of laboratory findings. Interdisciplinary collaboration will be vital, as the convergence of biology, medicine, and computational science is key to driving innovation in neuroscience. Collectively, multi-omics offers a transformative lens for studying nerve injury and regeneration, strengthening our grasp of underlying mechanisms and laying the groundwork for tailored, effective interventions. Future research should continue to harness the vast capabilities of multi-omics while integrating diverse findings to propel scientific progress and translate discovery into meaningful clinical outcomes in this dynamic and promising field.

## Additional files:

***Additional Table 1:*** *Multiomics studies identifying biomarkers of nervous system injury and repair published from 2020–2025.*

***Additional Table 2:*** *Key signaling pathways in neural regeneration and their multi-omics evidence.*

***Additional Table 3:*** *Comparison of regenerative features between CNS and PNS after injury.*

## Data Availability

*All relevant data are within the paper and its Additional files.*
